# Current Advances in Epidemiology, Diagnosis, and Management of TAVR-Associated Infective Endocarditis: A Narrative Review

**DOI:** 10.31083/RCM44599

**Published:** 2026-01-22

**Authors:** Zhenzhen Li, Dawei Lin, Jianing Fan, Jiaxin Miao, Wenzhi Pan, Daxin Zhou

**Affiliations:** ^1^School of Health Science and Engineering, University of Shanghai for Science and Technology, 200093 Shanghai, China; ^2^Department of Cardiology, Zhongshan Hospital, Fudan University, Shanghai Institute of Cardiovascular Diseases, 200032 Shanghai, China; ^3^National Clinical Research Center for Interventional Medicine, 200032 Shanghai, China

**Keywords:** transcatheter aortic valve replacement, infective endocarditis, TAVR-IE, epidemiology, diagnosis, microbiology, risk factors, antibiotic prophylaxis, clinical outcomes

## Abstract

With ongoing technological advancements and device innovations, transcatheter aortic valve replacement (TAVR) has become a well-established therapeutic approach for managing aortic stenosis and regurgitation. As indications for TAVR expand, particularly into younger patient populations, the incidence of TAVR-associated infective endocarditis (TAVR-IE) has concurrently increased. Although the reported incidence of TAVR-IE remains relatively low (0.3%–2.0% per 100 patient-years), its clinical outcomes are notably poor, with mortality rates considerably higher than those observed in general infective endocarditis (IE). Moreover, the microbiological profile of TAVR-IE differs distinctly from surgical aortic valve replacement-associated IE (SAVR-IE), predominantly involving *Enterococcus* spp., *Staphylococcus aureus*, and coagulase-negative staphylococci. This review systematically summarizes the epidemiology, diagnosis, microbial etiology, prevention strategies, clinical prognosis, and management approaches for TAVR-IE, providing clinical insights and identifying key areas for future research.

## 1. Introduction

Since its introduction, transcatheter aortic valve replacement (TAVR) has emerged as a safe and effective therapeutic option for patients with aortic valve disease, primarily due to its minimally invasive nature and expedited postoperative recovery. With ongoing refinement of techniques, advancements in device technology, and broad clinical adoption, TAVR utilization has continued to rise. Global procedural volumes have exhibited sustained double-digit annual growth over the past decade, surpassing 250,000 implants in 2023 alone [1]. Moreover, the clinical indications for TAVR have expanded beyond its initial application in high-surgical-risk patients deemed unsuitable for conventional surgery to include lower-risk and increasingly younger populations [2].

Infective endocarditis (IE) remains a rare but severe complication of TAVR, consistently associated with poor clinical outcomes. Although its incidence is relatively low (0.3%–2.0% per 100 patient-years [3, 4, 5, 6]), the absolute burden of TAVR-associated IE (TAVR-IE) is increasing in parallel with growing procedural volumes and broader use in younger patients [6, 7]. Typical clinical presentations include fever and new-onset heart failure, both of which significantly impair quality of life [8]. Severe TAVR-IE can lead to life-threatening complications such as acute heart failure, renal failure, septic shock, myocardial infarction, and systemic embolization, each posing significant therapeutic challenges and contributing to elevated mortality rates [7, 9]. Compared to general IE (in-hospital mortality: 15%–30% [10, 11]; 1-year mortality: ~40% [12, 13]), TAVR-IE carries a notably worse prognosis, with reported in-hospital mortality ranging from 16% to 64% and 1-year mortality reaching 27%–75% [3, 4, 5, 6], exceeding rates observed in both general IE and post-surgical endocarditis.

The convergence of rising TAVR utilization and the dire clinical consequences of TAVR-IE highlights the need for this comprehensive review. Here, we systematically synthesize current evidence on the epidemiology, diagnosis, microbiology, prevention, outcomes, and management of TAVR-IE, with the aim of enhancing clinical recognition and informing evidence-based practice.

## 2. Epidemiological Features of Post-TAVR Infective Endocarditis

Despite significant advancements, including iterative device innovation, increasing operator proficiency, streamlined procedural workflows, and expanding indications to include intermediate- and low-risk patients, the reported overall incidence of IE following TAVR remains between 0.3 and 2.0 per 100 patient-years. While surgical aortic valve replacement (SAVR) is inherently more invasive, multiple studies have demonstrated comparable overall IE rates between SAVR and TAVR, with no significant differences observed in in-hospital, early (≤1 year), late (>1 year), or overall IE incidence across treatment cohorts [4, 9, 14, 15, 16].

However, subgroup analyses suggest differential risk profiles across specific patient populations. One study reported a higher overall IE risk among intermediate-risk patients undergoing TAVR (2.3%) compared to those receiving SAVR (1.2%), although this difference narrowly missed statistical significance (odds ratio: 1.92; 95% CI: 0.99–3.72; *p* = 0.05; *I*^2^ = 0%) [17]. Conversely, an analysis of a comprehensive UK national database revealed a significantly higher 60-month cumulative incidence of IE following SAVR (2.4% [95% CI: 2.3–2.5]) compared to TAVR (1.5% [95% CI: 1.3–1.8]; hazard ratio: 1.60; *p* < 0.001) [18]. Supporting a potentially lower TAVR-associated risk, pooled data from three randomized trials reported by Lanz *et al*. [19] showed a modestly reduced cumulative IE incidence following TAVR (1.01% [95% CI: 0.47%–1.96%]) relative to SAVR (1.58% [95% CI: 0.97%–2.46%]; *p* = 0.047). Collectively, these findings suggest that although overall IE incidence is broadly comparable between TAVR and SAVR, the risk of post-TAVR IE may vary across patient subgroups and surgical risk strata.

The temporal distribution of IE following TAVR also demonstrates distinct patterns. In a longitudinal study of low-risk TAVR patients, cumulative IE incidence was 0% in the very early phase (≤30 days), 1.5% in the early phase (31–365 days), and 2.8% in the late phase (>1 year) post-procedure [20]. Similarly, the Swiss TAVR Registry reported a markedly higher incidence of early IE (1.48 per 100 person-years) compared to late IE (0.40 per 100 person-years) [3]. The highest risk period occurs within the first 100 days post-TAVR, with an incidence reaching 2.6 per 100 person-years [3]. A national multicenter cohort study further showed that early IE accounted for 64% of all post-TAVR IE cases [21], aligning with prior reports that the majority of TAVR-IE episodes occur within the first postoperative year [20, 21, 22, 23]. These findings underscore the critical importance of optimizing perioperative and early postoperative management to mitigate the risk of TAVR-IE.

## 3. Etiology and Pathogenesis

The principal portals of entry for TAVR-IE include soft tissue infections and intravascular access sites [22]. Unlike the microbial patterns observed in native valve endocarditis (NVE) or prosthetic valve endocarditis following surgical replacement (SAVR-IE), TAVR-IE exhibits a distinct pathogen profile. *Enterococcus* species, *Staphylococcus aureus*, and coagulase-negative staphylococci (CoNS) represent the predominant causative organisms [14, 21, 22]. Notably, compared with SAVR-IE, *Staphylococcus aureus* is more frequently implicated in TAVR-IE, whereas streptococcal infections are less common.

*Enterococcus* species are among the leading etiological agents of TAVR-IE [22, 23, 24], with significantly greater prevalence than in NVE or SAVR-IE [25]. This association may be related to the widespread use of the transfemoral approach in TAVR, as *Enterococcus* spp. preferentially colonize warm, moist regions such as the groin [26]. The second most prevalent pathogen is *Staphylococcus aureus*, which exhibits a higher incidence in TAVR-IE compared to SAVR-IE [7]. Critically, *S. aureus* infection in this context is associated with markedly increased virulence, conferring nearly double the in-hospital mortality (47.8% vs. 26.9%) and 2-year mortality (71.5% vs. 49.6%) relative to IE caused by other pathogens [15, 27, 28]. The increased incidence of *S. aureus* may be attributable to frequent invasive procedures (e.g., hemodialysis, intravenous access) in post-TAVR patients, which may compromise integumentary or mucosal barriers and elevate the risk of bacteremia [25]. CoNS rank as the third most common etiological agents, accounting for over 15% of TAVR-IE cases in several observational cohorts [22, 23, 29]. In contrast, streptococcal species, though still implicated, are significantly less common in TAVR-IE than in SAVR-IE (6.9% vs. 21%) [15, 22, 28]. Importantly, the temporal distribution of pathogens varies: *Enterococcus faecalis* predominates in very early (≤30 days) and early (31–365 days) TAVR-IE, whereas *S. aureus* and streptococcal species are more frequently isolated in late (>1 year) infections.

Less common pathogens include Gram-negative (GN) bacteria and fungi. GN bacteremia-associated TAVR-IE may occur in up to 5% of cases, with a notably earlier onset (median time: 1.1 months post-implantation) compared to other etiologies [4]. This abbreviated latency strongly suggests periprocedural contamination of the implanted device. Furthermore, the groin region, a frequent site of transfemoral access, may be colonized by multidrug-resistant GN organisms [30], potentially contributing to this risk.

## 4. Risk Factors

Multiple factors are significantly associated with an increased risk of infective endocarditis following TAVR. As observed in broader IE epidemiology [31], male sex consistently correlates with a higher risk of TAVR-IE [4, 5, 18, 21, 22]. The association with age is more complex: although advanced age is a known risk factor for IE in general, several studies have paradoxically identified younger age as an independent risk factor for TAVR-IE [22, 28]. This apparent contradiction may be attributed to the higher burden of severe comorbidities in younger patients selected for TAVR over surgical valve replacement [22, 28, 32]. Several comorbidities and clinical conditions have been shown to substantially increase the risk of TAVR-IE, with relative risk elevations ranging from 39% to 71%, including: renal impairment, chronic lung disease, history of infective endocarditis, permanent pacemaker implantation, diabetes mellitus, prior atrial fibrillation, intravenous drug use, heart failure, liver disease [14, 15, 22, 33, 34] (Table 1, Ref. [4, 5, 7, 16, 17, 18, 19, 22, 23, 28, 34, 35]). TAVR-related procedural factors also contribute to increased TAVR-IE risk. These include moderate or greater residual aortic regurgitation, low prosthetic valve position, vascular and bleeding complications, absence of balloon pre-dilation, valve-in-valve procedures, and progressive increases in transvalvular peak pressure gradients [4, 18, 21, 22]. However, no statistically significant difference in TAVR-IE incidence has been observed between balloon-expandable and self-expanding valves [5, 22]. The access route has also been identified as a potential determinant. A national registry study reported a higher risk of TAVR-IE associated with transapical or transsternal approaches compared to the transfemoral access [5, 14, 27, 36]. Notably, *Enterococcus* species are frequently isolated in TAVR-IE cases following transfemoral access, likely reflecting groin colonization and catheter-related contamination [14, 22, 23].

**Table 1.** **Incidence, microbiology, correlative factor, and outcomes across main studies of IE after TAVR**.

First author	Incidence of IE	Microbiology	Risk factors for TAVR-IE	TAVR-IE mortality
Tinica G [4]	0.3–2.0 per 100 person-years	*Streptococci* (25.3%), *staphylococcus* (25.3%), *enterococci* (24.1%)	Male, intubated, new pacemaker implantation IE and CKD	38.3%
Wang J [5]	0.9 per 100 person-years	*Enterococci* (24.3%), *Staphylococcus aureus* (22.7%)	Male, endotracheal intubation, moderate to severe residual aortic regurgitation, perioperative peripheral artery disease	In-hospital: 37.8%
Harding D [7]	0.2–3.1 per 100 person-years	*Staphylococcus aureus*, *enterococci*, coagulase negative staphylococcus	Younger, male, CKD, diabetes, chronic obstructive pulmonary disease, peripheral artery disease, moderate aortic regurgitation, valve in valve (ViV), self-expanding CoreValve	In-hospital: 36%–64%
	2–6.2 at 5 years			
Cahill TJ [18]	3.57 per 1000 person-years	*Enterococci* (25.9%), oral streptococci (16.4%), *S. aureus* (11.8%)	Younger, male, atrial fibrillation, dialysis	1 year: 45.6%
	1.5 at 5 years			
Ando T [17]	2.0 per 100 person-years	*Staphylococcus aureus*, *enterococci*	Younger, diabetes, moderate to severe aortic regurgitation, male, hospital infection	NA
Kolte D [28]	1.7 per 100 person-years	*Staphylococcus* (30.4%), streptococci (29.9%), *enterococci* (20.5%)	Younger, history of heart failure requiring a permanent pacemaker, in-hospital cardiac arrest, major bleeding, sepsis	In-hospital: 15.6%
Regueiro A [22]	1.1 per 100 person-years	*Enterococci* (24.6%), *Staphylococcus aureus* (23.3%), CNS (16.8%)	Younger, male, history of diabetes mellitus, moderate to severe residual aortic regurgitation	In-hospital: 36.0%
				2 years: 66.7%
Fauchier L [16]	1.89 per 100 person-years	*Streptococci* (29.0%), *enterococci* (22.7%), *Staphylococcus aureus* (15.8%), CNS (13.2%)	Men, frailty index, atrial fibrillation, anaemia	1 year: 32.8%
Butt JH [34]	1.6 per 100 person-years		Male, CKD	In-hospital: 20.9%
	5.8 at 5 years			1 year: 40.0%
Amat-Santos IJ [35]	0.50 at 1 year	CNS (24.5%), *Staphylococcus aureus* (20.8%), *enterococci* (20.8%), oral streptococci (5.7%)	NA	In-hospital: 47.2%
				1 year: 66.0%
Del Val D [23]	5.92 per 1000 person-years	*Enterococci* (25.1%), *Staphylococcus aureus* (24.0%), CNS (18.2%)	Acute postoperative renal injury	In-hospital: 32.0%
				1 year: 46.6%
Lanz J [19]	2.47 per 1000 person-years	*Streptococci* (38.5%), *enterococci* (23.1%), *Staphylococcus aureus* (15.4%), CNS (15.4%)	Diabetes, heart failure	1 year: 27.3%
	1.01 at 5 years			

TAVR, transcatheter aortic valve replacement; IE, Infective endocarditis; TAVR-IE, TAVR-associated IE; CKD, chronic kidney disease; CNS, coagulase-negative staphylococci; NA, not available.

Anatomical characteristics of the native aortic valve also influence TAVR-IE risk. Specifically, a higher calcific burden and elevated transvalvular gradients have been associated with early-onset infections. Bjursten *et al*. [33] demonstrated that each 1-mmHg increase in baseline mean transaortic gradient corresponded to a 2% increase in relative risk for early TAVR-IE among patients with severe valvular calcification. Importantly, this association was limited to early infections, with no significant correlation found for late-onset cases. These findings support the importance of targeted perioperative antibiotic prophylaxis, particularly during the early postoperative period when endothelial healing is incomplete [4, 22].

## 5. Presentation and Diagnosis

Establishing a definitive diagnosis of TAVR-IE is more challenging than diagnosing NVE. Clinical presentations are often atypical, particularly during the early post-procedural period, when fever and systemic inflammatory responses can occur even in the absence of true infection [37]. Fever is the most common symptom of TAVR-IE, followed by new-onset heart failure, occurring in approximately 80% and 40% of cases, respectively [4, 22, 37].

Echocardiography, especially transesophageal echocardiography (TEE), plays a central role in the diagnosis and assessment of NVE. However, the microbiological spectrum of TAVR-IE is more diverse than that of native valve endocarditis [38, 39]. In the context of TAVR, both transthoracic echocardiography (TTE) and TEE are frequently limited by acoustic shadowing artifacts and poor intra-stent visualization. As a result, TTE has significantly lower diagnostic utility for TAVR-IE compared to NVE [40]. The combined sensitivity of TTE and TEE for diagnosing TAVR-IE is approximately 67.8%, compared with 73% for prosthetic valve endocarditis (PVE) following surgery and 89.9% for NVE [22, 40].

Although echocardiography remains the cornerstone of imaging-based diagnosis, several advanced imaging modalities, including multidetector computed tomography (MDCT), cardiac magnetic resonance imaging (CMR), and ^18^F-fluorodeoxyglucose positron emission tomography (^18^F-FDG PET), have become indispensable adjuncts. These modalities offer improved visualization of intracardiac anatomy and superior structural resolution. Multimodal imaging has been shown to enhance diagnostic accuracy, particularly in detecting endocardial involvement and extracardiac complications with greater sensitivity [41, 42]. A retrospective multicenter analysis reported that ^18^F-FDG PET/computed tomography (PET/CT) led to diagnostic reclassification in 33% of patients initially evaluated using the Duke criteria, reinforcing its clinical utility in suspected TAVR-IE [43]. In 2015, the European Society of Cardiology (ESC) incorporated ^18^F-FDG PET and MDCT findings into the diagnostic algorithm for suspected IE, recognizing them as key imaging criteria in surgical decision-making [44, 45]. The ESC guidelines recommend performing FDG-PET/CT within 3 months after cardiac surgery to reduce false positives due to postoperative inflammation [46, 47, 48].

A study by San *et al*. [49] found a relatively low positivity rate (23%) for FDG-PET/CT performed 1 month post-TAVR. Interestingly, although FDG uptake intensity did not significantly differ between controls and confirmed TAVR-IE cases, distinct uptake patterns were observed. While control patients showed circumferential or semicircular uptake, TAVR-IE cases exhibited focal or multifocal uptake, localized to the central or ventricular portions of the anterior stent segment of the prosthetic valve. These findings suggest that FDG-PET/CT is a reliable diagnostic tool for TAVR-IE when performed at least 1 month after valve implantation. Meanwhile, MDCT, routinely employed pre-procedurally for anatomical assessment, also provides high-resolution imaging of the coronary vasculature and perivalvular complications (e.g., abscesses), offering superior visualization compared to TTE [50, 51].

## 6. Management and Clinical Outcomes

Antibiotic therapy remains the cornerstone of medical management for patients with TAVR-IE. In the largest observational cohort to date (n = 250), most patients (50.4%) received β-lactam antibiotics in combination with another antibiotic class; 15.2% were treated with β-lactams alone, and 21.2% received vancomycin either as monotherapy or in combination with another agent [22]. However, current antibiotic recommendations for TAVR-IE are largely extrapolated from existing PVE guidelines [44, 52]. The ESC provides specific antibiotic regimen recommendations:

Early PVE (≤1 year post-implantation): Vancomycin (30 mg/kg/day IV, divided into two doses), gentamicin (3 mg/kg/day IV or IM, single dose), and rifampin (added after 3–5 days to target dormant bacteria).

Late PVE (>1 year post-implantation): In penicillin-allergic patients: Vancomycin and gentamicin remain the first-line combination. In penicillin-tolerant patients: Vancomycin may be replaced with ampicillin (12 g/day IV, divided into 4–6 doses) in combination with flucloxacillin or piperacillin.

We identified 12 observational studies assessing outcomes in patients with TAVR-IE. Reported in-hospital mortality ranged from 15.6% to 63.6%, while 1-year mortality varied between 40.0% and 60% (Table 1). Several studies [15, 22, 29, 53] identified heart failure, sepsis or septic shock, chronic hemodialysis or chronic kidney disease, acute renal failure during hospitalization, and elevated EuroSCORE as independent predictors of in-hospital mortality. Regueiro *et al*. [22] evaluated causes of death among patients who survived hospitalization for TAVR-IE (n = 160). During a median follow-up of 10.5 months, 50 patients died. The most common causes of death included infection-related complications (n = 14), sudden death (n = 8), cardiovascular causes (n = 5), cancer (n = 3), other causes (n = 5), and unknown causes (n = 15).

Acute heart failure, acute renal failure, septic shock, acute myocardial infarction, and systemic embolism are among the most common complications associated with TAVR-IE during hospitalization. Additionally, the incidence of periannular aortic abscesses detected in patients diagnosed with endocarditis following TAVR ranges from 3.6% to 19.1% [15, 28, 33, 54], whereas rates reported in patients who underwent surgical aortic valve replacement (SAVR) vary from 30% to 55% [40, 55, 56]. Surgical intervention is primarily indicated in cases of IE-induced valvular dysfunction leading to acute heart failure, perivalvular infection causing annular or aortic root abscesses, destructive penetrating lesions of vessels and/or myocardium, new-onset atrioventricular block, or persistent bacteremia [57]. However, across various studies, antibiotic therapy alone remains the most common strategy, even in the presence of severe complications [21, 23, 28, 33, 54, 58, 59]. Previous research indicates that although 80% of TAVR-IE patients have surgical indications, the actual rate of surgical intervention is exceedingly low [15, 19, 22]. Notably, compared to medical therapy alone, surgery has not been associated with improved in-hospital mortality, 30-day readmission rates, or one-year all-cause mortality [60, 61, 62]. To date, no specific recommendations have been established for surgical management in this population, and indications are often individualized based on local expertise.

Isolated involvement of the TAVR prosthesis is the most common presentation (48%), such as perivalvular abscesses, pseudoaneurysms, or vegetations on the valve surface that impair normal function [60, 63]. However, nearly one-third of TAVR-IE patients present with IE involving at least two cardiac structures, including the mitral valve, cardiac devices, or right-sided IE [60, 63]. The low sensitivity of echocardiography in PVE is well recognized [44, 54], and other imaging techniques such as multidetector computed tomography and ^18^F-FDG PET/CT are valuable in suspected PVE and have been incorporated into recent guidelines [64].

Meanwhile, neurological events, particularly stroke, remain among the most common and potentially disabling complications associated with TAVR-IE, often involving the mitral valve. The incidence of stroke during hospitalization for post-TAVR IE is approximately 10% [65]. Methicillin-resistant *Staphylococcus aureus* (MRSA) infection is more common among TAVR-IE patients who experience stroke (37.5% vs. 15.1%). Additionally, stroke patients exhibit higher one-year overall mortality (66.3% vs. 45.6%). During the initial hospitalization for IE, 25% of stroke patients underwent surgical treatment; however, compared to non-surgical management, surgery did not improve long-term outcomes in Stroke-IE patients [65, 66].

## 7. Prevention of TAVR-IE

Preventing TAVR-IE remains a critical aspect of postprocedural management in patients undergoing transcatheter aortic valve replacement. Although the optimal antibiotic regimen for prophylaxis remains uncertain, most consensus guidelines recommend perioperative antibiotic administration. The ESC advises antimicrobial prophylaxis with a first-generation cephalosporin, initiated within 1 h before the procedure and continued for up to 48 h post-TAVR (Class IIa recommendation) [44]. In contrast, the American Heart Association (AHA) and the Centers for Disease Control and Prevention (CDC) recommend a single preoperative antibiotic dose (Class I recommendation). If this dose is inadvertently omitted, administration within 2 h postoperatively is considered acceptable [54, 67]. However, considerable variability in antibiotic regimens and dosing frequency exists among centers [55], reflecting the absence of standardized protocols in this domain. Notably, in 2017, the AHA downgraded the level of evidence supporting antibiotic prophylaxis for IE in TAVR recipients from Class B (moderate-quality evidence) to Class C-LD (limited data) [68]. Of particular concern, a large registry-based analysis involving 7203 patients reported that nearly 50% of perioperative (<100 days) TAVR-IE cases were caused by microorganisms resistant to the most commonly employed prophylactic regimens [3]. In response, the International Society of Cardiovascular Infectious Diseases (ISCVID) recommends the administration of enterococcus-active agents within 60 min before arterial puncture: amoxicillin–clavulanate or ampicillin–sulbactam for patients without penicillin allergy, and vancomycin for those with penicillin hypersensitivity. A second dose is advised if the procedure exceeds 2 h [9].

Approximately half of all TAVR-IE cases are classified as healthcare-associated IE, representing more than twice the incidence of procedure-related IE [22, 29]. This disparity may stem from the increased frequency of healthcare exposures and interventions in TAVR recipients, many of which are associated with transient bacteremia and elevated IE risk [40]. As such, some experts advocate minimizing non-essential medical procedures that may predispose patients to bloodstream infections.

Finally, unlike dental procedures, the role of antibiotic prophylaxis for invasive interventions involving the respiratory, gastrointestinal, genitourinary, or cutaneous systems has been increasingly questioned [9]. This shift is primarily driven by concerns regarding antimicrobial resistance, the risk of adverse drug events, and the high incidence of unnecessary treatment. As a result, widespread antibiotic prophylaxis for non-dental procedures is no longer routinely recommended. Instead, the development of prosthetic valve systems incorporating novel antimicrobial biomaterials offers a promising strategy to reduce the incidence of bacteremia and prosthesis-related infections.

## 8. Future Expectations

Although the incidence of post–TAVR-IE remains relatively low, the exponential increase in TAVR procedures, particularly among lower-risk and younger patients, is expected to substantially expand the population at risk for this life-threatening complication. Advances in device technology, reductions in procedural invasiveness, improved operator proficiency, and optimized perioperative care may collectively help mitigate the incidence of early TAVR-IE.

In parallel, standardized and precise imaging protocols should be adopted, incorporating not only TTE but also early TEE in all suspected cases of IE. In patients with inconclusive echocardiographic findings yet high clinical suspicion, advanced imaging modalities such as ^18^F-fluorodeoxyglucose positron emission tomography/computed tomography (FDG-PET/CT) or single-photon emission computed tomography/computed tomography (SPECT/CT) should be employed to support or exclude the diagnosis of TAVR-IE.

These coordinated efforts are essential to facilitate the development of dedicated, evidence-based TAVR-IE management guidelines, derived from high-quality, procedure-specific data rather than extrapolations from SAVR literature.

## 9. Conclusions

The incidence of TAVR-IE ranges from 0.3% to 2% per 100 person-years across most studies. *Enterococcus*, *Staphylococcus aureus*, and coagulase-negative staphylococci are the predominant causative organisms. Although mortality estimates vary, clinical outcomes remain poor, with in-hospital mortality reported between 15.6% and 63.6% and 1-year mortality ranging from 40.0% to 60%. TAVR-IE demands heightened clinical vigilance. Standardized procedural protocols, thorough preoperative assessment, and appropriate perioperative antibiotic prophylaxis constitute essential preventive strategies. Transesophageal echocardiography remains central to early detection, while the development of antimicrobial biomaterial-coated prosthetic valves represents a promising avenue for future risk reduction.

## Abbreviations

TAVR, transcatheter aortic valve replacement; TAVR-IE, TAVR-associated infective endocarditis; SAVR, surgical aortic valve replacement; SAVR-IE, SAVR-associated infective endocarditis; IE, infective endocarditis; NVE, native valve endocarditis; CoNS, coagulase-negative staphylococci; TTE, transthoracic echocardiography; TEE, transesophageal echocardiography; MDCT, multi-detector computed tomography; CMR, cardiac magnetic resonance imaging; ^18^F-FDG PET/CT, fluorine-18 fluorodeoxyglucose positron emission tomography/computed tomography; ESC, European Society of Cardiology; AHA, American Heart Association; CDC, Centers for Disease Control and Prevention; ISCVID, International Society of Cardiovascular Infectious Diseases; EuroSCORE, European System for Cardiac Operative Risk Evaluation; SPECT/CT, single-photon emission computed tomography/computed tomography; GN, Gram-negative.
